# Supplementary material for: The Systems Biology Research Tool: evolvable open-source software
Source: BMC Syst Biol. 2008 Jun 29;2:55. doi: 10.1186/1752-0509-2-55 (PMC2446383; doi:10.1186/1752-0509-2-55)
Supplement: Additional file 1 — SBRT Archive. An archive of the current version of the Systems Biology Research Tool. [file 1752-0509-2-55-S1.zip › sbrt-1.4.0/doc/developers_guide/api/index-files/index-14.html]

N-Index


|  |  |  |
| --- | --- | --- |
| **Overview** \| Package \| Class \| Use \| **Tree** \| **Deprecated** \| **Index** \| **Help** | |  |
| **PREV LETTER**   **NEXT LETTER** | **FRAMES**    **NO FRAMES**     **All Classes** |  |


A B C D E F G H I J K L M N O P Q R S T U V W X Y Z 

---


## **N**

**nameClassMap** - Static variable in class sbrt.shell.ProgramSolverFactory: The correspondence between the strings used to denote a particular program solver and the name of the actual class it represents. **neighbors(Object)** - Method in class sbrt.kernel.math.graph\_theory.DirectedGraph: Returns the number of nodes neighboring the provided node. **NetworkInfoManager** - Class in sbrt.shell.mng.fba: This class is used to manage fluxome information experiments. **NetworkInfoManager()** - Constructor for class sbrt.shell.mng.fba.NetworkInfoManager: **NetworkInfoProcess** - Class in sbrt.kernel.fba.proc: This class is used to gather basic information about a fluxome. **NetworkInfoProcess(CatalyzedFluxome)** - Constructor for class sbrt.kernel.fba.proc.NetworkInfoProcess: Constructs a new flux activity experiment. **NetworkInfoProcess.Results** - Class in sbrt.kernel.fba.proc: This class is used to represent the information gathered about a fluxome. **NEVER\_ACTIVE\_FILE\_NAME** - Static variable in class sbrt.shell.mng.fba.FluxPlasticityManager: The keyword used to denote the name of the output file for the names of never-active reactions. **neverActive()** - Method in class sbrt.kernel.fba.proc.FluxPlasticityProcess.Results: Returns the number of fluxes that are never active. **next()** - Method in class sbrt.kernel.math.geom.comp.CdHarComputer: Computes and returns the next random interior point of the convex polytope. **next(int)** - Method in class sbrt.kernel.math.geom.comp.CdHarComputer: Computes the specified number of random interior points, and returns the last one computed. **next()** - Method in class sbrt.kernel.util.UnmodifiableIterator: See Iterator.next(). **next()** - Method in class sbrt.shell.io.ComiFileSource: Returns the next column-oriented map input file with the next name returned by the file name source. **next()** - Method in class sbrt.shell.io.ComoFileSource: Returns the next column-oriented map output file with the next name returned by the file name source. **next()** - Method in class sbrt.shell.io.fba.CofoofSource: Returns the next column-oriented map output file with the next name returned by the file name source. **next()** - Method in class sbrt.shell.io.fba.RofoofSource: Returns the next column-oriented map output file with the next name returned by the file name source. **next()** - Method in class sbrt.shell.io.fba.SfoofSource: Returns the next column-oriented map output file with the next name returned by the file name source. **next()** - Method in class sbrt.shell.io.RomiFileSource: Returns the next row-oriented map input file with the next name returned by the file name source. **next()** - Method in class sbrt.shell.io.RomoFileSource: Returns the next row-oriented map output file with the next name returned by the file name source. **next()** - Method in class sbrt.shell.io.SerializedFileIterator: Returns the next object in the file. **next()** - Method in class sbrt.shell.io.SmoFileSource: Returns the next column-oriented map output file with the next name returned by the file name source. **next()** - Method in class sbrt.shell.io.TextFileLineIterator: Returns the next element in the file. **nextObject()** - Method in class sbrt.shell.io.SerializedFileIterator: Returns the next object in the file. **nodes()** - Method in class sbrt.kernel.math.graph\_theory.DirectedGraph: Returns the number of nodes in this graph. **nodes()** - Method in class sbrt.kernel.math.graph\_theory.Path: Returns the number of nodes in this path. **NonEmptyStringFormat** - Class in sbrt.shell.text: This class is used to define a format for `String` objects. **NonEmptyStringFormat()** - Constructor for class sbrt.shell.text.NonEmptyStringFormat: **NormalDist** - Class in sbrt.kernel.math.stat: This class is used to represent normal distributions. **NormalDist(double, double)** - Constructor for class sbrt.kernel.math.stat.NormalDist: Constructs a new normal distribution with the provided average and standard deviation. **nullCheck(Object)** - Static method in class sbrt.kernel.util.Util: Ensures the provided object is not null. **NUMBER\_OF\_ON\_FLUXES** - Static variable in class sbrt.shell.mng.fba.RandomConstraintsManager: The keyword used to denote the number of active fluxes.

---


|  |  |  |
| --- | --- | --- |
| **Overview** \| Package \| Class \| Use \| **Tree** \| **Deprecated** \| **Index** \| **Help** | |  |
| **PREV LETTER**   **NEXT LETTER** | **FRAMES**    **NO FRAMES**     **All Classes** |  |


A B C D E F G H I J K L M N O P Q R S T U V W X Y Z 

---
